# Conservation of Local Honeybees (*Apis mellifera* L.) in Southeastern Turkey: A Preliminary Study for Morphological Characterization and Determination of Colony Performance

**DOI:** 10.3390/ani13132194

**Published:** 2023-07-04

**Authors:** Atilla Oztokmak, Gonca Ozmen Ozbakir, Oznur Çaglar

**Affiliations:** 1 Ankara Provincial Directorate of Agriculture and Forestry, 06560 Ankara, Turkey; atilla.oztokmak@tarimorman.gov.tr; 2 Department of Animal Science, Faculty of Agriculture, University of Harran, 63300 Şanlıurfa, Turkey; 3 Adıyaman Nuts Research Institute Directorate, 02040 Adıyaman, Turkey; oznur.caglar@tarimorman.gov.tr

**Keywords:** *Apis mellifera*, local honeybees, conservation, morphology, queen rearing, colony strength

## Abstract

**Simple Summary:**

The biggest obstacles in the conservation of existing honeybee subspecies and ecotypes may be the concentration of beekeeping activity in certain flora zones as well as the beekeeper’s choice of queen genotype. In this study, various morphological characteristics and colony performance parameters of local honeybees in Southeastern Turkey were investigated. As a result, homogenous honeybees can be found in the Adıyaman province in terms of morphological characteristics. Although the colony performance values of local honeybees are modest, their capacity to adapt to dry regions and limited vegetation and the preservation of their genetic material are critical attributes. Local honeybees can be used as genetic material in selection and breeding due to their hygienic behavior, survival rate, and high wintering abilities.

**Abstract:**

The aim of this study was to investigate the morphological traits and colony performance parameters of local honeybees of the Adıyaman province for future breeding programs. The study was carried out in 2019–2020; a total of 39 colonies were obtained from 13 apiaries in 5 districts, which represented local honeybees, and they were placed in an isolated area. At the same time, 835 worker bees representing local honeybees were assessed for 21 morphological features. There was a difference between the district groups according to the morphological traits (*p* < 0.05). In terms of the morphological characteristics of honeybees, the correct classification rate to their original groups was determined to be 65.1%. The difference between the district groups was statistically significant regarding the number of frames covered with bees, brood area, and hygienic behavior (*p* < 0.05). The average weight at the emergence of queens according to district groups and flight activity was found to be significant according to the periods (*p* < 0.01). As a result, homogeneous honeybees can be found in provinces when considering morphological characteristics. Although colony performance values are low, it is essential to protect the bees’ genetic material given their ability to adapt to arid climates and insufficient flora.

## 1. Introduction

Honeybees are social insects that live in colonies with ecological, evolutionary, and economic importance, and they are referred to as superorganisms [1,2,3]. Although Turkey has the honeybee subspecies of the Caucasian (*Apis mellifera caucasica*), Syrian (*Apis mellifera syriaca*), Iranian (*Apis mellifera meda*), and Anatolian honeybees (*Apis mellifera anatoliaca*), which have adapted to different geographical and climatic conditions, it has also various ecotypes that are morphologically and genetically defined [3,4,5,6,7,8,9]. The morphological characteristics are mostly used in the definition and classification of honeybee subspecies and ecotypes, and there have been attempts to determine the distinctive features of honeybee populations [3]. Wing geometric morphometrics and molecular methods are recent techniques that also provide a similar discrimination of honeybee subspecies. The geometric morphometrics method is only used for wing characteristics; it is a practical method, but molecular techniques are expensive and require trained personnel [10,11,12,13,14,15,16,17].

Performance tests are conducted in honeybee colonies to determine the colony’s strength and productivity. While parameters such as brood area, number of frames covered with bees, and flight activity express the strength of the colony, parameters such as honey yield express the productivity. Colony strength should express the population size in a colony as well as the number of next-generation worker bees in open and sealed broods. Measurements such as brood area (cm^2^), percentage of capped brood cells, total bee weight (kg), honey weight, and disease prevalence and presence have been examined by various researchers to determine colony strength [18,19,20,21]. All parameters, including the robbing [22,23], hygienic behavior [24,25], and swarming tendency [26,27] of the colonies, depend on the genetic structure of the queen and the drones that she mates with. Climatic and environmental factors, also including beekeeper applications, are among the elements that directly affect colony performance [1,20]. The adaptation of honeybees to their environment is explained by the annual growth pattern of the colony, the host–parasite balance, and the nutritional sources, and all of these factors interact with the environmental changes [28]. As pollinators, honeybees are an important component of global biodiversity, which provides wild plants and essential ecosystem services to crops [29]. Protecting honeybee diversity and supporting local breeding studies should be given top priority to prevent the losses of the colony and optimize sustainable productivity [30]. The importance of honeybee subspecies and ecotypes that are specific to different regions, as well as their conservation studies, is still an ongoing priority [31,32,33].

To define the populations of the honeybees that are indigenous to regions, their morphological characteristics are first determined, followed by the explanation of their behavior and performance parameters in their colonies; the variations are revealed by measurements that are taken at different times. In this way, their suitability for conservation and breeding programs is examined. Geometric morphometry and molecular markers that support or validate classical morphometric measurements can likewise be utilized for this purpose. In this study, we aimed to define the morphological characteristics of the Adıyaman honeybees, which have not been examined in detail before. At the same time, we also aimed to examine the behavior and performance characteristics of the colonies formed, with the queens that were reared in the colonies purchased from apiaries representing the region. This is the first study carried out in the province with respect to these objectives.

## 2. Materials and Methods

Within the scope of the study, the apiaries representing local honeybees (*Apis mellifera* L.) were determined using trips that scanned for them in the districts of Adıyaman, which is located in the southeast of Turkey. The honeybee colonies were purchased from the apiaries in districts where there has been stationary beekeeping for years, where they do not purchase queens, and where they are not located on migratory beekeeping routes. In addition, worker bee samples were obtained to measure their morphological characteristics. A total of 39 colonies were purchased from 5 districts that met these criteria, and morphological characteristics were determined from 43 colonies. The measurement of the morphological traits was performed using a Leica S8APO stereomicroscope at an appropriate magnification. The measurement of the following traits was performed according to Ruttner (1988) [3] on at least 15 worker bees from each colony: tongue length (TL) (mm), length of hairs on tergite 5 (HL) (mm), forewing length (mm) (FWL), forewing width (FWW) (mm), length of cubital vein a (CVA) (mm), length of cubital vein b (CVB) (mm), width of tomentum (a) (mm), width of the dark stripe (b) (mm), tergite 3 longitudinal (T3) (mm), tergite 4 longitudinal (T4) (mm), femur length (FeL) (mm), tibia length (TiL) (mm), and metatarsus length (MetL) and width (MetW) (mm). The secondary characters calculated from the main characters were as follows: T3 + T4 longitudinal (T3 + T4) (mm), hind leg length (HLL) (mm), forewing index (FWI), tomentum index (TI), cubital index (CI), cubital index percentage (CI%), and metatarsal index (MTI). The detailed information about the locations of the apiary where the colonies were purchased and the samples of the workers were obtained for the morphological measurements are stated in Table 1.

Three colonies were purchased from each apiary in 2019. These colonies were moved to the village of İncebağ, where there was no resident beekeeper in the east, west, and north and which is surrounded by the Atatürk Dam Lake in the south. This location as an isolated area has been restricted to the entrance of the beekeepers. The queens were reared from one larvae source colony, representing each district group. Thirty one-day-old larvae were used for grafting for each group. We aimed to carry out the performance tests in 75 trial colonies with at least 15 in each group and with the newly-reared queens being randomly selected. The queens were naturally mated. The measurement of performance parameters in the trial colonies and the queen rearing process were repeated in 2019 and 2020. The acceptance rate of larvae and weight at the emergence and preoviposition periods were determined in the queens reared. In the colonies formed with these queens, the colony survival rate, winter colony losses, brood area, number of frames covered with bees, flight activity, defensive behavior, swarming tendency, robbing, hygienic behavior, and honey yield were examined [20,25,26,34,35]. The performance tests were carried out sixty days after the oviposition of the queens in each trial colony. The starter colonies for each district group comprised 65 support colonies from local honeybees; mating nuclei were used, the trial colonies (5-frame nuc hives) were built as 3 frames of equal strength (honey, pollen, sealed brood), and a 1:1 sugar syrup was used for feeding.

For each sampled location group, the univariate analysis of variance and multivariate analysis of variance methods were used on 21 of the morphological variables. The univariate analysis of variance was used to compare the groups in terms of the colony performance parameters. The multiple comparisons of the group means were conducted using the Duncan test with respect to the morphological characteristics and performance parameters. The relationships between the morphological characteristics were determined using the Pearson correlation coefficient. The stepwise discriminant analysis was applied to determine the power of the morphological characteristics in separating the groups. All data were analyzed using IBM SPSS Statistics v. 21 software.

## 3. Results

### 3.1. The Morphological Characteristics

The average tongue length of the Adıyaman honeybees was determined to be 6.471 mm. The average tongue length was significant according to the district groups. (*p* < 0.001). The tongue length was the highest in the Besni group and the lowest in the Samsat group. According to the multiple comparisons test, the group mean of the Kahta and Tut groups were found to be similar. While the overall average length of hairs was 0.197 mm, the highest average (0.213 mm) was obtained in the Kahta group and lowest average (0.189 mm) was obtained in the Besni group. In terms of the average length of hairs, the Merkez and Tut groups were similar.

There was also a significant difference between the groups in terms of the forewing index, forewing length, forewing width, and the length of cubital vein a and b (*p* < 0.05). The average of the forewing length and width were determined to be 8.789 mm and 3.109 mm, respectively. While the Merkez group was the lowest value obtained (41.43) and the Kahta group (46.45) was the highest value obtained in terms of cubital index (%) values, similar values were obtained for the Kahta and Tut groups. It was found that there was a difference (*p* < 0.05) between the groups in terms of the hindleg morphological characteristics, where the general average of the hindleg length was 7.788 mm. The general average of T3G + T4G, which expresses the body size of worker bees, was 4.387 mm. The descriptive statistics for the morphological characteristics of the worker bees from the five district groups in Adıyaman are presented in Table 2.

As expected, there were high and significant positive correlations between the various body parts of the workers. There are significant prominent correlations within the morphological characteristics of the Adıyaman honeybees, such as between the tongue length and the forewing length (r = 0.761; *p* < 0.01), between the tongue length and the forewing width (r = 0.694; *p* < 0.01), and between the tongue length and the hindleg length (r = 0.490; *p* < 0.01). Significant correlations were found between the forewing length and the hindleg length (r = 0.583; *p* < 0.01), between the forewing width and the hindleg length (r = 0.553; *p* < 0.01), and between the tergite 3 longitudinal and the hindleg length (r = 0.576; *p* < 0.01).

In terms of the morphological characteristics, the multivariate analysis of variance and stepwise discriminant analyses were applied according to the colony averages in the district groups. Wilk’s Lambda test statistic was found to be significant (*p* < 0.001). In the characteristics of the tongue length, forewing length, forewing width, length of cubital vein a, width of tomentum, tergite 3 longitudinal, length of tibia, metatarsus length and width, and the leg length, the difference between the district groups was significant (*p* < 0.05). The information for the groups examined in the discriminant analysis is given in Table 3.

As a result of the discriminant analysis, the first canonical function explains 87.5% of the total variance, and the second function explains 12.5% of the remaining variance (Table 4). According to the first two canonical discriminant functions, the standardized coefficients for the contributions of the variables to the functions are given in Table 5. As a result of the analysis, it was found that 14 morphological characteristics significantly contributed to the model. According to the structure matrix, while the first canonical discriminant function was found to be highly correlated with the variables of the width tomentum, length of cubital vein a, and tergite 4 longitudinal, other variables were found to be highly correlated with the second canonical discriminant function. The characteristic of the tongue length had the highest canonical correlation in the second function.

**Figure 1:**
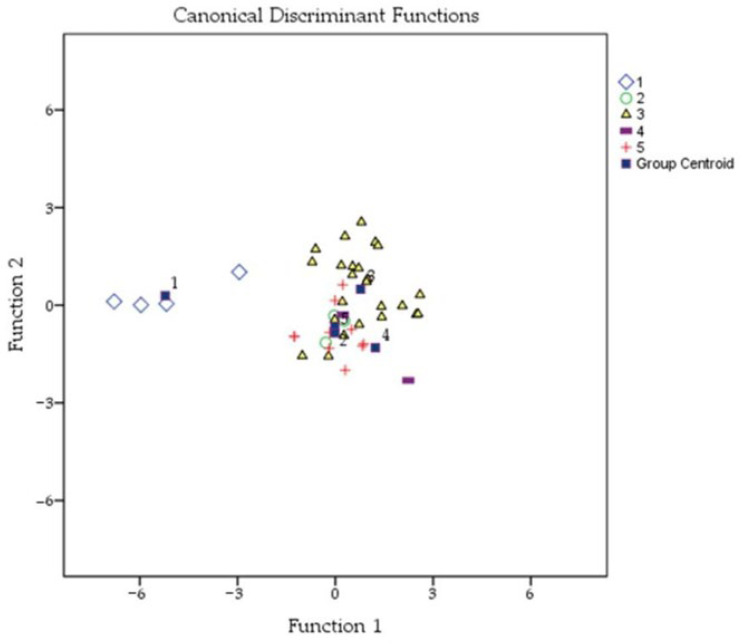
Scatter diagram of local honeybees according to district groups.

In terms of the morphological characteristics, the correct classification rate of the honeybees to their original groups was determined to be 65.1%. According to the obtained discriminant functions, the calculated group memberships with respect to the morphological characteristics are given in Table 6. Only the honeybee samples of the Besni group were included in its own group at a rate of 100%. Meanwhile, the honeybees of Kahta and Merkez were correctly classified in their own groups at a rate of 66.7%, the honeybees of Samsat and Tut were classified at a rate of 50%. As can be seen in the scattering diagram (Figure 1), the group centroids of Kahta and Tut are very close according to the canonical discriminant functions. The distribution of the Merkez honeybee group compared with the Kahta, Samsat, and Tut groups is also observed. It can be stated that the honeybees sampled from Besni show more uniform distribution in terms of the morphological characteristics in their groups.

### 3.2. Queen Rearing

A total of 150 larvae, with 30 being selected from each source colony to represent the district groups, were grafted. The acceptance rate of larvae was determined to be 90% for 2019 and 91% for 2020. While the highest acceptance rate of larvae in 2019 was achieved in Tut, Kahta, and Besni district groups, the lowest rate was achieved in the Merkez group. In 2020, the highest acceptance rate of larvae was obtained at a rate of 93% in the Kahta and Besni district groups, while the lowest rate was obtained at a rate of 90% in the Merkez, Samsat, and Tut district groups. In the trial colonies, the preoviposition time of queens was determined to be 13 days on average. 

According to the weight at the emergence of the queens, it was determined that the Kahta (175.93 mg) and Merkez groups (171.34 mg) had the highest averages, and they had similar averages; the Samsat (165.75 mg) and Tut groups (166.94 mg) were also similar. The lowest average weight at the emergence of the queen was in the Besni (159.91 ± 6.946 mg) group. It was determined that there was an interaction between the years and the district groups in terms of the weight at the emergence of the queen (*p* < 0.05). While the general average weight at the emergence of the queens reared in 2019 was 160.87 ± 10.292 mg, the average weight at the emergence of the queens reared in 2020 was 174.91 ± 10.718 mg. According to this result, it was observed that the variation between the years was significant in terms of the average weight at the emergence of the queens (*p* < 0.01); additionally, the difference in average weight at the emergence of the queens according to district groups was found to be significant (*p* < 0.01). Among the district groups, the highest average weight at the emergence of the queen was observed in the Kâhta group with 178.13 ± 9.133 mg in 2020. The lowest average weight at the emergence of the queen was weighed at 169.06 ± 13.102 mg for the Besni group in the same year (Table 7).

### 3.3. Colony Performance Parameters

The measurements for the number of frames covered with bees and brood area were taken in four periods in 2020. The variation between the district groups was significant with respect to the number of frames covered with bees (*p* < 0.05). The mean number of frames covered with bees in the Samsat group was the highest at 4.22, and the averages of the other groups were also found to be similar. The general average number of frames covered with bees was 3.98. In 2020, the highest brood area was measured to be 1466.77 ± 133.420 cm^2^ in the first period, and the lowest one was 175.91 ± 40.918 cm^2^ in the fourth period. In terms of the period general averages in the district groups, the highest brood area was determined to be 1086.91 ± 105,733 cm^2^ in the Tut group, while the lowest was determined to be 895.28 ± 96.727 cm^2^ in the Merkez group. The averages of the brood area in the Kahta and Tut groups were similar (Table 8).

The flight activity was calculated by the number of the worker bees that took flight for 1 min in front of the hive before noon in four periods in 2020. There was no difference in terms of the flight activity in 2019 (*p* > 0.05). In terms of the flight activity in 2019, the highest average was measured in the Merkez group (10.50 ± 4.500) and the lowest was measured in the Samsat group (6.42 ± 1.645). The flight activity was found to be significant according to the periods in 2020 (*p* < 0.01). As a result of the Duncan test, the flight activity of the first and second periods were high and were different from the third and fourth periods. It was observed that the period with the highest flight efficiency was the first period (6 August 2020), with an average of 19.08 ± 3.691. The lowest average was measured to be 2.00 ± 0.466 in the fourth period (7 October 2020). Among the district groups, the highest flight efficiency was measured in the Merkez group with an average of 13.72 ± 1.850, and the lowest flight efficiency was measured in the Samsat group with an average of 9.45 ± 1.318.

A black suede ball (5 × 4 cm) was used to determine the aggressiveness in the trial colonies. The number of stings on the ball, numbers of the workers following the ball, and workers waiting at the hive entrance were determined. In terms of the sting numbers, the difference between the groups was not significant (*p* > 0.05). Among the groups, it was observed that the Besni group had the highest sting numbers with an average of 4.21 ± 0.352; Samsat had the lowest sting numbers with an average of 1.77 ± 0.387. In terms of the behavior of tracking the ball, it was determined that the Besni group had the highest average with a value of 5.08 ± 0.849, and the Tut group was the lowest with a value of 2.50 ± 0.437 among the district groups (*p* < 0.05). In terms of the behavior of tracking the ball according to the periods, the second period (27 August 2020) was the highest at 4.24, and the fourth period (7 October 2020) was the lowest at 2.18 (*p* < 0.05). During the ball test, the difference was not significant in the periods and groups in terms of the number of workers waiting at the hive entrance. The general average of the number of workers waiting at the hive entrance was 3.06.

The hygienic behavior of each trial colony was assessed using the pin test (100 closed cells in the brood area), and the cleaned cells in the area were counted after 24 h. The difference between the district groups was found to be significant in 2020 in terms of the hygienic behavior (*p* < 0.05). The lowest value was found in the Samsat group (73.50%), and the highest value was found in the Kahta group (86.67%); there were similar rates in the Besni (85.85%), Kahta, Merkez (85.71%), and Tut (83.3%) groups.

There was no difference between the district groups in terms of the averages of the honey yield in 2019 (*p* > 0.05). In 2019, the Merkez group had the highest average honey yield (3.0 kg), while the Tut group had the lowest average yield (2.17 kg). In terms of the honey yield in 2020, there was no difference between the district groups (*p* > 0.05). It was observed that the highest honey yield average in 2020 was in the Samsat group with a value of 3.97 ± 0.423 kg. The lowest averages, which were also similar averages, were measured in the Besni, Kahta, and Tut district groups. When 2019 and 2020 were examined, it was found that there was a difference between the years in terms of honey yield (*p* < 0.01). While the general average of the honey yield in the district groups in 2019 was 2.471 ± 0.194 kg, it was determined to be 3.76 ± 0.113 kg in 2020. 

There were high colony losses during the first year of the experiment. The colony survival rates in 2019 were determined to be 33%, 26%, 13%, 47%, and 40% for the Besni, Kahta, Merkez, Samsat, and Tut district groups, respectively. The colony survival rates of the Besni, Kahta, Central, Samsat, and Tut district groups in 2020 were determined to be 93%, 86%, 86%, 64%, and 71%, respectively. Winter colony losses were estimated as the ratio between the number of surviving colonies in spring to the number of colonies in the previous autumn. In terms of the winter colony losses, values of 83% in the Tut group and 100% in the other groups was obtained in the district groups in 2019. In 2020, an 85% overwintering success was obtained in the Tut group, 90% in the Samsat district group, and 100% in the Merkez, Besni, and Kahta groups. It was observed that there was no swarming tendency or robbing behavior in the trial colonies in both years.

## 4. Discussion

To prevent the genetic variation losses, optimize the sustainable productivity, and ensure continuous adaptation to the environmental changes, the diversity of the honeybee should be protected, local breeding studies should be prioritized, and the phenotypic diversity for honeybee should be determined for the protection of local honeybees [36]. The intense mixture of honeybee populations on a global scale is mostly attributed to the common migratory beekeeping practices and the replacement of queens, as well as colonies with nonnative breeds or hybrids of different subspecies [37]. One of the most widely used methods for the protection of honeybees as both pollinators and honey producers is the evaluation of the morphometric characteristics when they are evaluated using appropriate general methods [38]. Honeybee samples that were obtained from some western provinces of Turkey 30 years ago and those collected in 2017 were compared with respect to geometric morphometry in a study [39]. As a result of that study, old and new bee samples were clustered into two main clusters. It is important to protect honeybee ecotypes and genetic resources in their original regions [40], especially in order to determine the effects of commercial queen use and migratory beekeeping on the morphological variation [41]. In this study, besides determining the morphological characteristics of the Adıyaman honeybee, which has not been studied in detail before, the parameters of the colony performance were also investigated. Therefore, the morphological characteristics are the markers that also show the change in the honeybee populations over time. However, the evaluation of the morphological data together with the genetic data will provide more useful results.

Morphological characteristics such as the tongue length and length of hairs in honeybees are explained by the adaptation to climate, topography, and flora, and these differ among subspecies [3]. In this study, the average tongue length of the Adıyaman honeybees was found to be 6.471 mm; it was very close to the tongue length (6.46 mm) of the Anatolian bee, *A. mellifera anatoliaca* [3]. Compared with the tongue length ((6.03 mm) [42] and (6.27 mm) [43]) for honey bees in the neighboring province of Şanlıurfa, it was high. The tongue length in other subspecies was reported as 6.33 mm for the Iranian bee, *A. mellifera meda*; 6.19 mm for the Syrian bee, *A. mellifera syriaca*; and 7.04 mm for the Caucasian bee, *A. mellifera caucasica* [3]. In another study on the Anatolian bee, the tongue length of Kırşehir (6.47 mm) and Beypazarı-1 (6.48 mm) bees were reported similarly to the results of this study [44]. Like the results of this study, the tongue length for the Anatolian bee was 6.48 mm [6]. However, the tongue lengths of the five groups examined in this study showed variation between 6.39–6.57 mm, and a variation was determined between the groups (*p* < 0.05). In the distinguishing of the Adıyaman honeybees according to their morphological characteristics, the tongue length was the characteristic with the highest canonical correlation in the second function. The Besni group honeybees were separated from the other groups. 

In this study, while the average length of the hair was 0.197 mm, it was found that the highest average was (0.213 mm) in the Kahta group and the lowest mean was (0.189 mm) in the Besni group. The hair length was reported as 0.29 mm for the Anatolian bee, 0.28 mm for the Iranian bee, 0.23 mm for the Syrian bee, and 0.33 mm for the Caucasian bee [3]. This is similar to the result of an old study [42] conducted in the neighboring province, but a higher hair length was determined according to the result of a more recent study [43]. The hair length of Syrian bees [45] was higher than Adıyaman honeybees. The hair length was reported s 0.19 mm in Kırşehir honeybees, 0.20 mm in Beypazarı-1 honeybees, 0.21 mm in the Beypazarı-2 honeybees, 0.20 mm in the Çankırı honeybees, 0.21 mm in Eskişehir honeybees, and 0.28 mm in the Caucasian honeybees [44]. In this study, the hair length was found to be similar to the hair length of Kırşehir and Beypazarı honeybees, which are defined as Anatolian honeybees.

The general average forewing length and forewing width of the Adıyaman honeybees were determined as 8.789 mm and 3.109 mm, respectively; it was higher than the forewing length and forewing width values reported for Şanlıurfa honeybees [43]. The forewing length was reported as 8.935 mm for the Adıyaman honeybees [46], and the general average of the forewing length for the Adıyaman honeybees was lower in this study. In this study, the forewing length and forewing width were different from the values determined in the Kırşehir and Beypazarı honeybees [44]; it was found to be lower than the forewing length and width for various genotype groups [47]. The cubital index in the Adıyaman honeybees varied between 2.29 and 2.48, and its general mean was found to be 2.40. It was higher than the cubital index reported for the Anatolian bee, but it was similar to the value reported for the Iranian bees [3]. It was also similar to the cubital index values (2.42) for Şanlıurfa honeybees [43], (2.36) Syrian bees [45], and (2.38) and Adıyaman honeybees [46]. 

In terms of the hindleg characteristics of the Adıyaman honeybees, there was a difference between the groups and the general average of the hindleg length, which was determined as 7.788 mm. The hindleg length determined in this study was similar to the values reported [3] for Iranian bees (7.821 mm) and Syrian bees (7.828 mm) and was higher than the value reported for Şanlıurfa honeybees [43]. Furthermore, the hindleg length was found to be similar to the Central Anatolian honeybee ecotypes and to Syrian honeybees [44,45,47]. The general average of the T3 + T4, which expresses the body size of worker bees, was 4.387 mm for the Adıyaman honeybees. In this study, the T3 + T4 value was similar to Iranian (4.356 mm) [3] and Syrian honeybees (4.35 mm) [48]. It was similar to the body size reported by [42,43] for Şanlıurfa honeybees. It was also compatible with the body size values reported for the Central Anatolian honeybee ecotypes [6,49]. The environment has an important effect on honeybee morphological characteristics, and it was previously reported that honeybee morphological traits correlate with colony productivity [50,51,52,53]. In this study, there were significant correlations between the tongue length and the forewing width, the forewing length and the hindleg length, and the forewing length and the hindleg length of Adıyaman honeybees. Different correlations were noticed for the same morphological characteristics in the Caucasian, Yığılca, and Korgan honeybee groups [54], and similar correlations were observed for the same morphological traits in the southeastern border honeybees of Turkey [43]. 

The weight at the emergence of the queen is affected by many factors, such as genotype, larval age, rearing season, condition of the foster colonies, and environmental conditions, and it shows variation [1,55,56,57]. For the Adıyaman honeybees, the weight at the emergence of the queen in 2020 varied between 169 and 175 mg; this result is compatible with the results reported by [58,59,60]. On the other hand, it is lower than the results reported by [61,62,63]. 

In the study, the brood area and number of the frames covered with bees for the Adıyaman honeybees were lower than the values reported for different genotypes in different regions [64,65,66]. There were variations in the Adıyaman honeybee groups in terms of the flight activity. The measurements in terms of both flight activity and other colony performance parameters were explained for the late periods of the beekeeping season. When examined according to similar periods of previous studies, the flight activity was compatible in similar ecological conditions [67], while it was lower than the results previously reported when in different ecological conditions [21,35]. In terms of the aggressiveness behavior, the Besni group (sting number) was found to have higher values than the others. When the studies in different genotypes and regions for the aggressiveness behavior were examined, it was compatible in similar ecological conditions [67], was lower than the Muğla ecotype, and was similar to Italian bees [68]; it was similar to the Anatolian and the Caucasian honeybees; and it was lower than the Yığılca honeybees [26]. The frozen brood or pin test are useful screening tools for the hygienic behavior. The freeze-killed brood test is the most reliable and conservative screening procedure for hygienic behavior [24,69]. The results of the hygienic behavior test determined in this study were compatible with the other results [66,67]. However, the pin test results for screening the hygienic behavior in our study may not be as relevant as other findings regarding hygienic behavior in honey bees. The honey yield was also low in the trial colonies of the Adıyaman honeybee, whose colony development was poor. The honey yield was lower than the values reported by [65,66,68].

## 5. Conclusions

Because beekeepers use queens from different subspecies in their colonies, the natural mating biology of the queen can lead to different levels of hybridization. The discriminant analysis results show that the honeybees sampled from Adıyaman are in a mixed form except for the Besni group because of their higher morphological values. For the Kahta, Merkez, Tut, and Samsat honeybees, which are distributed in a cluster, the group centroids of the Kahta and Tut honeybees are especially closer to each other, while the group centroids of the Merkez and Samsat are farther from each other; however, these groups are overlapping. As a general conclusion, it is possible to find uniform honeybee samples in Adıyaman, and advanced studies should be carried out with more observation numbers and sampling taking place from different regions of the province. Because this study included a detailed examination of the morphological characteristics of the Adıyaman honeybees, it will become possible to determine the morphological variation at the level of years and districts. A confirmation of the results of classical morphometric measurements using geometric morphometry or genetic analyses will provide clearer and more useful results.

The second part of the study was the determination of performance parameters in the colonies of the Adıyaman honeybee. Sufficient measurement data could not be obtained in 2019 because the queen bee was expected to form her own progeny group after the determination of where the districts that were determined as the colonies of the Adıyaman honeybee were located, the purchase, and the queen rearing process. When the data of 2020 were examined in general, it was observed that the weight at the emergence of the queen was low. In the trial colonies, three frames of worker bees were initially equalized with honey, pollen, and sealed brood, and the development was low, which was in line with the number of frames covered with bees and brood area during the season. Therefore, the honey yield was found to be low. The study area is a region that has arid and hot climate characteristics and does not have a significant diversity and richness in terms of flora. Because the colonies’ hygienic behavior, survival rate, and overwintering abilities were high and there was no swarming tendency or robbing, the Adıyaman honeybees could be used as local genetic material in selection and breeding. Brood rearing and food shortage in regions with short and hot winters and long and dry summers negatively affect the development and productivity of colonies; however, local honeybee genotypes that adapt to the arid conditions, especially in light of global climate change, will gain more importance.

## Figures and Tables

**Table 1:** Locations where workers were sampled and where the colonies were purchased.

District	Village	Colony Purchased Apiaries	Altitude	Coordinates	District	Village	Colony Purchased Apiaries	Altitude	Coordinates
Besni	Akyazı	3	520	37°31′11″ N 38°06′26″ E	Merkez	Karahöyük	3	646	37°43′29″ N 38°24′36″ E
Besni	Gümüşlü	3	608	37°39′09″ N 38°04′43″ E	Merkez	Uludam	3	676	37°45′04″ N 38°27′31″ E
Kâhta	Fıstıklı	-	779	37°39′58″ N 38°41′53″ E	Merkez	Mal Pınarı	3	521	37°29′12″ N 38°10′06″ E
Kâhta	Bejyan	-	779	37°40′27″ N 38°41′30″ E	Merkez	Kuyulu	3	514	37°29′37″ N 38°12′19″ E
Kâhta	Susuz	3	570	37°38′25″ N 38°44′12″ E	Samsat	Taşkuyu	3	621	37°36′58″ N 38°30′06″ E
Merkez	Ilıcak	3	549	37°34′27″ N 38°08′01″ E	Tut	Köseli	3	728	37°46′00″ N 38°02′31″ E
Merkez	Karakoç	3	554	37°39′98″ N 38°12′37″ E	Tut	Köseli	-	705	37°46′35″ N 38°02′50″ E
Merkez	Ovakuyucak	3	532	37°40′20″ N 38°18′26″ E	Tut	Elçiler	3	629	37°46′44″ N 38°04′00″ E

**Table 2:** Descriptive statistics of morphological characteristics by district groups (mean ± SEM).

Characters	Besni *n* = 79	Kâhta *n* = 60	Merkez *n* = 480	Samsat *n* = 40	Tut *n* =176	General *n* = 835
TL (mm)	6.578 ± 0.0132 ^a^	6.390 ± 0.0175 ^c^	6.517 ± 0.0085 ^b^	6.293 ± 0.0277 ^d^	6.367 ± 0.0111 ^c^	6.471 ± 0.0110
HL (mm)	0.189 ± 0.0127 ^c^	0.213 ± 0.0020 ^a^	0.196 ± 0.0009 ^bc^	0.205 ± 0.0033 ^ab^	0.196 ± 0.0014 ^bc^	0.197 ± 0.0023
FWL (mm)	9.002 ± 0.0199 ^a^	8.558 ± 0.0178 ^d^	8.845 ± 0.0120 ^b^	8.634 ± 0.0320 ^c^	8.656 ± 0.0141 ^c^	8.789 ± 0.0145
FWW (mm)	3.175 ± 0.0095 ^a^	3.035 ± 0.0094 ^d^	3.127 ± 0.0046 ^b^	3.061 ± 0.0147 ^dc^	3.067 ± 0.0054 ^c^	3.109 ± 0.0060
CVA (mm)	0.528 ± 0.0046 ^cd^	0.549 ± 0.0048 ^ab^	0.549 ± 0.0023 ^ab^	0.498 ± 0.0069 ^d^	0.528 ± 0.0033 ^cd^	0.540 ± 0.0031
CVB (mm)	0.235 ± 0.0029 ^ab^	0.240 ± 0.0023 ^a^	0.225 ± 0.0015 ^b^	0.229 ± 0.0038 ^b^	0.230 ± 0.0015 ^b^	0.228 ± 0.0018
a (mm)	0.986 ± 0.0058 ^c^	1.034 ± 0.0076 ^b^	1.059 ± 0.0019 ^a^	1.039 ± 0.0038 ^b^	1.033 ± 0.0026 ^b^	1.044 ± 0.0029
b (mm)	0.218 ± 0.0049 ^bc^	0.218 ± 0.0042 ^bc^	0.247 ± 0.0019 ^a^	0.255 ± 0.0074 ^a^	0.246 ± 0.0046 ^a^	0.244 ± 0.0031
T3 (mm)	2.168 ± 0.0066 ^c^	2.192 ± 0.0068 ^b^	2.232 ± 0.0030 ^a^	2.190 ± 0.0091 ^b^	2.182 ± 0.0051 ^cb^	2.210 ± 0.0043
T4 (mm)	2.112 ± 0.0061 ^d^	2.139 ± 0.0070 ^bc^	2.178 ± 0.0036 ^a^	2.139 ± 0.0115 ^bc^	2.226 ± 0.1044 ^a^	2.176 ± 0.0257
FeL (mm)	2.531 ± 0.0056 ^b^	2.536 ± 0.0068 ^b^	2.569 ± 0.0035 ^a^	2.562 ± 0.0127 ^a^	2.537 ± 0.0052 ^b^	2.555 ± 0.0047
TiL (mm)	3.125 ± 0.0085 ^b^	3.120 ± 0.0093 ^b^	3.207 ± 0.0046 ^a^	3.197 ± 0.0148 ^a^	3.143 ± 0.0062 ^b^	3.178 ± 0.0061
MetL (mm)	2.022 ± 0.0055 ^b^	2.017 ± 0.0082 ^b^	2.074 ± 0.0033 ^a^	2.055 ± 0.0099 ^a^	2.026 ± 0.0062 ^b^	2.053 ± 0.0047
MetW (mm)	1.156 ± 0.0040 ^b^	1.148 ± 0.0054 ^d^	1.182 ± 0.0020 ^a^	1.155 ± 0.0060 ^cd^	1.155 ± 0.0025 ^cd^	1.169 ± 0.0027
HLL (mm)	7.677 ± 0.0169 ^b^	7.673 ± 0.0203 ^b^	7.849 ± 0.0100 ^a^	7.814 ± 0.0348 ^a^	7.706 ± 0.0150 ^b^	7.788 ± 0.0136
T3 + T4 (mm)	4.279 ± 0.0123 ^a^	4.330 ± 0.0135 ^a^	4.409 ± 0.0061 ^a^	4.328 ± 0.0202 ^a^	4.408 ± 0.1041 ^a^	4.387 ± 0.0285
FWI	35.27 ± 0.000 ^a^	35.45 ± 0.000 ^a^	35.35 ± 0.000 ^a^	35.45 ± 0.001 ^a^	35.43 ± 0.000 ^a^	35.37 ± 0.000
CI	2.292 ± 0.0441 ^b^	2.290 ± 0.028 ^b^	2.485 ± 0.0194 ^a^	2.208 ± 0.0561 ^b^	2.315 ± 0.0240 ^b^	2.404 ± 0.0251
CI%	44.79 ± 0.787 ^ba^	43.89 ± 0.535 ^b^	41.43 ± 0.345 ^c^	46.45 ± 1.198 ^a^	44.00 ± 0.449 ^b^	42.71 ± 0.463
TI	4.15 ± 0.086 ^b^	4.83 ± 0.080 ^a^	4.41 ± 0.035 ^b^	4.21 ± 0.134 ^b^	4.36 ± 0.058 ^b^	4.39 ± 0.053
MTI	57.17 ± 0.200 ^a^	56.95 ± 0.266 ^a^	56.99 ± 0.086 ^a^	56.23 ± 0.256 ^b^	57.09 ± 0.201 ^a^	56.89 ± 0.142

^a,b,c,d^; different letters in the same column represent different means (*p* < 0.05).

**Table 3:** Information about the groups examined in multivariate analysis.

Location	Besni	Kâhta	Merkez	Samsat	Tut	General
Workers (*n*)	79	60	480	40	176	835
Colonies (*n*)	4	3	24	2	10	43

**Table 4:** Eigenvalues and variance explanation rates of canonical discriminant functions.

Function	Eigenvalue	% of Variance	Cumulative %	Canonical Correlation
1	3.332	87.5	87.5	0.877
2	0.476	12.5	100	0.568

**Table 5:** Structure matrix.

Characters	Function	
	1	2
b	−0.184	0.081
CVA	0.173	0.037
T4G	0.120	0.116
TL	−0.116	0.993
FWL	−0.189	0.850
FWW	−0.182	0.807
FeL	0.082	0.785
a	0.642	0.767
TiL	−0.138	0.699
T3G	−0.023	0.668
MetL	0.045	0.629
MetW	−0.085	0.620
HL	0.068	0.461
CVB	0.008	0.039

**Table 6:** Predicted group memberships of colonies in terms of morphological characteristics (*n*, %).

Original Group	Predicted Group Membership					
	Besni	Kahta	Merkez	Samsat	Tut	*n*, %
Besni	4 100					4 100
Kahta		2 66.7			1 33.3	3 100
Merkez		2 8.3	16 66.7	3 12.5	3 12.5	24 100
Samsat		1 50		1 50		2 100
Tut		1 10	1 10	3 30	5 50	10 100

Classification rate to original groups: 65.1%.

**Table 7:** Mean weight at the emergence of queens (mg).

District Groups	2019		2020		General	
	*n*	Mean ± SEM	*n*	Mean ± SEM	*n*	Mean ± SEM
Besni	16	150.75 ± 12.641	16	169.06 ± 13.102	32	159.91 ± 6.946 ^c^
Kâhta	15	173.73 ± 14.250	15	178.13 ± 9.133	30	175.93 ± 4.870 ^a^
Merkez	16	166.25 ± 7.742	16	176.44 ± 9.099	32	171.34 ± 4.834 ^ab^
Samsat	16	157.75 ± 10.266	16	173.75 ± 12.036	32	165.75 ± 0.771 ^b^
Tut	16	156.69 ± 6.809	16	177.19 ± 10.121	32	166.94 ± 0.771 ^b^
General	79	160.87 ± 10.292	79	174.91 ± 10.718	158	167.87 ± 0.352

^a,b,c^: different letters in the same column represent statistically different means (*p* < 0.05).

**Table 8:** Brood area (cm^2^).

Groups	1. Period (6 August 2020)		2. Period (27 August 2020)		3. Period (17 September 2020)		4. Period (7 October 2020)		General	
	*n*	Brood Area	*n*	Brood Area	*n*	Brood Area	*n*	Brood Area	*n*	Brood Area
Besni	13	1427 ± 124.6	13	1291 ± 95.6	13	473 ± 63.7	3	126 ± 64.6	42	997 ± 92.5 ^ab^
Kâhta	12	1687 ± 70.0	12	1275 ± 98.4	12	432 ± 45.8	2	234 ± 10.2	38	1084 ± 68.1 ^a^
Merkez	14	1339 ± 144.0	14	1120 ± 117.6	13	297 ± 36.8	2	93 ± 16.4	43	895 ± 96.7 ^b^
Samsat	10	1398 ± 213.8	9	1243 ± 157.0	9	591 ± 50.5	3	286 ± 67.5	31	1011 ± 129.7 ^b^
Tut	12	1493 ± 126.9	12	1180 ± 127.9	12	674 ± 69.7	1	39 ± 0.0	37	1086 ± 105.7 ^a^
General	61	1466 ± 133.4 ^a^	60	1219 ± 116.9 ^b^	59	485 ± 53.3 ^c^	11	175 ± 40.9 ^d^	191	1011 ± 97.2

^a,b,c,d^: different letters in the same column and row represent statistically different means (*p* < 0.05).

## Data Availability

Not applicable.
